# Bis(*N*-nitroso-*N*-pentyl­hydroxy­laminato-κ^2^ *O*,*O*′)copper(II)

**DOI:** 10.1107/S1600536814004978

**Published:** 2014-03-19

**Authors:** Ali Sheikh Bostanabad, Olga Kovalchukova, Svetlana Strashnova, Adam Stash, Igor Zyuzin

**Affiliations:** a Peoples’ Friendship University of Russia, 6 Miklukho-Mallaya, 117198 Moscow, Russian Federation; b Karpov Institute of Physical Chemistry, 10 Vorontsovo Pole, 105064 Moscow, Russian Federation; c The Institute of Problems of Chemical Physics of the Russian Academy of Sciences (IPCP RAS), Academician Semenov Avenue 1, Chernogolovka, Moscow region, 142432, Russian Federation

## Abstract

In the centrosymmetric title compound, [Cu(C_5_H_11_N_2_O_2_)_2_], the Cu^2+^ ion, located on an inversion centre (Wyckoff position 2*b*), is in a square-planar environment, surounded by four O atoms of the N—O groups of two *N*-nitroso-*N*-pentyl­hydroxy­laminate ligands [Cu—O = 1.9042 (17) and 1.9095 (16) Å]. The hy­droxy­laminate monoanions are bidentate chelating ligands. The Cu^2+^ cations form stacks along [010], with inter­molecular Cu⋯N contacts of 3.146 (2) and 3.653 (2) Å.

## Related literature   

The basic procedure for the synthesis of the reported complex is described by Zyuzin *et al.* (1997 ▶). For related structures of copper complexes with the *N*-nitroso­hydroxyl­amine derivatives, see: Abraham *et al.* (1987 ▶); Kovalchukova *et al.* (2013 ▶, 2014 ▶). The synthesis and properties of other metal nitroso­hydroxy­laminates are given in: Ahmed *et al.* (1988 ▶); Basson *et al.* (1992 ▶); Bolboaca *et al.* (2000 ▶); Kovalchukova *et al.* (2013 ▶); Najafi *et al.* (2011 ▶); Okabe & Tamaki (1995 ▶); Parkanyi *et al.* (1999 ▶); Pavel *et al.* (2000 ▶); Tamaki & Okabe (1998 ▶); Van der Helm *et al.* (1965 ▶). For applications of *N*-nitroso­hydroxyl­amine derivatives see: Lundell & Knowles (1920 ▶); Buscarons & Canela (1974 ▶); Oztekin & Erim (2000 ▶); McGill *et al.* (2000 ▶).
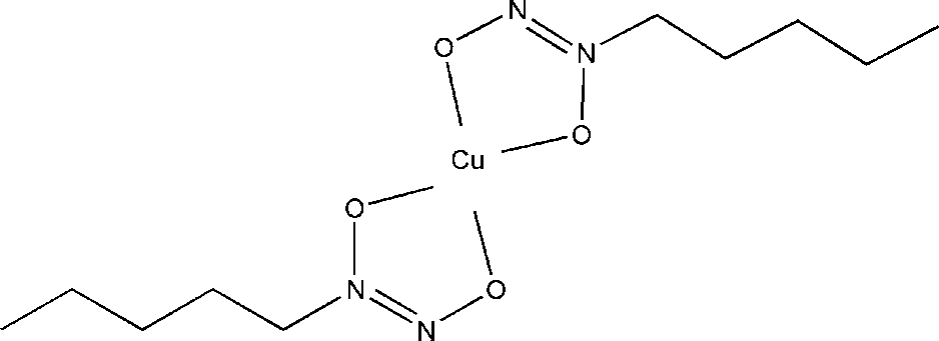


## Experimental   

### 

#### Crystal data  

[Cu(C_5_H_11_N_2_O_2_)_2_]
*M* *_r_* = 325.86
Monoclinic, 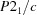

*a* = 14.325 (3) Å
*b* = 4.776 (1) Å
*c* = 11.619 (2) Å
β = 103.82 (3)°
*V* = 771.9 (3) Å^3^
*Z* = 2
Mo *K*α radiation
μ = 1.43 mm^−1^
*T* = 293 K
0.80 × 0.20 × 0.03 mm

#### Data collection  

Enraf–Nonius CAD-4 diffractometer
Absorption correction: part of the refinement model (Δ*F*) (Walker & Stuart, 1983 ▶) *T*_min_ = 0.202, *T*_max_ = 0.670
1509 measured reflections
1429 independent reflections
871 reflections with *I* > 2σ(*I*)
*R*_int_ = 0.030
3 standard reflections every 60 min intensity decay: none

#### Refinement  

*R*[*F* ^2^ > 2σ(*F* ^2^)] = 0.026
*wR*(*F* ^2^) = 0.073
*S* = 0.88
1429 reflections
88 parameters
H-atom parameters constrained
Δρ_max_ = 0.32 e Å^−3^
Δρ_min_ = −0.68 e Å^−3^

### 

Data collection: *CAD-4-PC* (Enraf–Nonius, 1993 ▶); cell refinement: *CAD-4-PC*; data reduction: *CAD-4-PC*; program(s) used to solve structure: *SHELXS97* (Sheldrick, 2008 ▶); program(s) used to refine structure: *SHELXL97* (Sheldrick, 2008 ▶); molecular graphics: *SHELXXTL* (Sheldrick, 2008 ▶); software used to prepare material for publication: *CIFTAB97* and *SHELXL97*.

## Supplementary Material

Crystal structure: contains datablock(s) I, global. DOI: 10.1107/S1600536814004978/pj2009sup1.cif

Structure factors: contains datablock(s) I. DOI: 10.1107/S1600536814004978/pj2009Isup2.hkl

Supporting information file. DOI: 10.1107/S1600536814004978/pj2009Isup3.mol

CCDC reference: 989916

Additional supporting information:  crystallographic information; 3D view; checkCIF report

## Figures and Tables

**Fig. 1.**
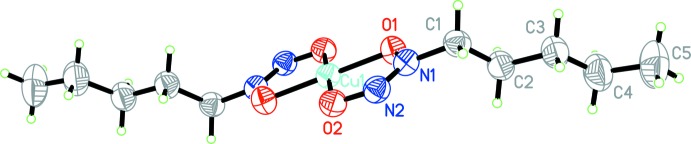
ORTEP view of C10H22CuN4O4 with atom labeling scheme (displacement ellipsoids are drawn at the 50% probability level for non-hydrogen atoms). The second half of the molecule is generated by the symmetry operator 1-x, 1-y,1-z.

**Fig. 2.**
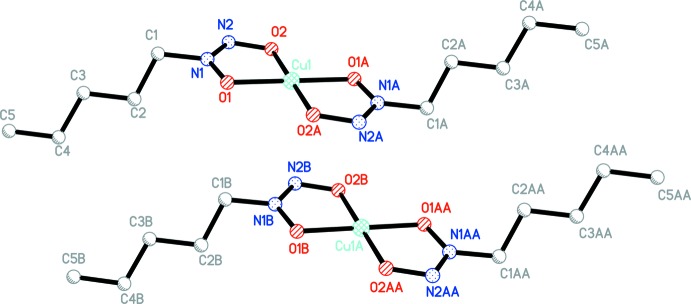
Mutual arrangement of neighboring complexes in a stack.

## supplementary crystallographic information

### 1. Comment

The chelate-forming derivatives of *N*-nitroso hydroxylamines form stable complexes with the metallic ions of various natures but only few of them have been structurally characterized (Abraham *et al.*, 1987; Ahmed *et al.*, 1988; Basson *et al.*, 1992; Bolboaca *et al.*, 2000; Kovalchukova *et al.*, 2013; Najafi *et al.*, 2011; Okabe & Tamaki, 1995; Parkanyi *et al.*, 1999; Pavel *et al.*, 2000; Tamaki & Okabe, 1998; Van der Helm *et al.*, 1965). Their ammonium and potassium salts are reported as good analytical reagents for different purposes (Lundell & Knowles, 1920; Buscarons & Canela, 1974; Oztekin & Erim, 2000). In addition, recently it was reported that many *o*-substituted *N*-nitroso-*N*-oxybenzenamines are good NO donors for both *in vitro* and *in vivo* assays (McGill *et al.*, 2000). The title compound C_10_H_22_CuN_4_O_4_ (Fig. 1) is centrosymmetric with the Cu^2+^ ion located on the inversion centre (Wyckoff position 2*b*) in square planar coordination, surounded by four oxo O atoms of the N—O groups of two organic ligands [Cu—O = 1.9042 (17) and 1.90905 (16) Å]. The molecule of the metal complex is completed by the 1-*x*, 1-*y*, 1-*z* symmetry operation. The mean deviation from the plane is 0.0199 Å. The *N*-nitroso-*N*-(n-pentyl)hydroxylaminate anions are bidentate chelating ligands. The Cu cations in the columns form stacks in the columns along the [010] direction with intermolecular Cu—N contacts equal to 3.146 (2) and 3.653 (2) Å (Fig. 2). The described coordination type of the central atom correlates with those described previously for the bis(N– nitroso-*N*-benzyl-hydroxylaminato-o,o) copper(II) (Kovalchukova *et al.*, 2013) and bis(*N*-nitroso-*N*-ethyl-hydroxylaminato-o,o) copper(II) (Kovalchukova *et al.*, 2014).

### 2. Experimental

The title compound was obtained in accordance with the previously published procedure (Zyuzin *et al.*, 1997) with some modifications. A solution of *n*-pentylmagnesium chloride was prepared from magnesium (12.2 g, 0.5 mol) and 1-bromopentane (75.5 g, 0.5 mol) in the dry Et_2_O (0.5 *L*). The NO gas was bubbled through the solution under vigorous stirring and cooling at such a rate that NO was almost entirely absorbed. The reaction mixture temperature was maintained in the range 248 to 243 K. After the period of a rapid NO absorption (1 h), stirring was continued in an NO atmosphere for 0.5 h until the NO absorption was completed, with a gradual increase in temperature to 263 K. The reaction mixture was purged with Ar, treated with MeOH (100 mL), poured into ice (300 g) and acidified with 2 *M* H_2_SO_4_. The organic layer was separated, and the aqueous layer was extracted with Et_2_O (100 mL × 3). The combined extracts were washed with 50 mL 1 *M* NaOH and 50 mL H2O. The aqueous layer was neutralized with 2 *M* H_2_SO_4_ until pH 4 and treated with 20 per cent CuSO_4_ solution (120 g, 0.2 mol). The blue precipitate was washed with water, dried and crystallized from EtOH. Yield 42.3 g (52 per cent), blue crystals, m.p. 355–356 K. Analysis calculated for C_10_H_22_CuN_4_O_4_: Cu 19.50; found: Cu 18.83. Single crystals of C_10_H_22_CuN_4_O_4_ were grown by the slow evaporation of the ethanol solution of the bis[*N*-nitroso-*N*-(*n*-pentyl)hydroxylaminato] copper(II) powdered sample.

### 3. Refinement

The structure of of C_10_H_22_CuN_4_O_4_ was solved by direct method and all non-hydrogen atoms were located and refined anisotropically. All the hydrogen atoms added using a riding model.

### Crystal data

**Table:**

[Cu(C_5_H_11_N_2_O_2_)_2_]	*F*(000) = 342
*M**_r_* = 325.86	*D*_x_ = 1.402 Mg m^−^^3^
Monoclinic, *P*2_1_/*c*	Mo *K*α radiation, λ = 0.71073 Å
Hall symbol: -P 2ybc	Cell parameters from 25 reflections
*a* = 14.325 (3) Å	θ = 9.7–11.9°
*b* = 4.776 (1) Å	µ = 1.43 mm^−^^1^
*c* = 11.619 (2) Å	*T* = 293 K
β = 103.82 (3)°	Plate, blue
*V* = 771.9 (3) Å^3^	0.80 × 0.20 × 0.03 mm
*Z* = 2	

### Data collection

**Table:**

Enraf–Nonius CAD-4 diffractometer	871 reflections with *I* > 2σ(*I*)
Radiation source: fine-focus sealed tube	*R*_int_ = 0.030
β-filter monochromator	θ_max_ = 25.5°, θ_min_ = 2.9°
ω/2τ scans	*h* = −17→16
Absorption correction: part of the refinement model (Δ*F*) (Walker & Stuart, 1983)	*k* = −5→0
*T*_min_ = 0.202, *T*_max_ = 0.670	*l* = 0→13
1509 measured reflections	3 standard reflections every 60 min
1429 independent reflections	intensity decay: none

### Refinement

**Table:**

Refinement on *F*^2^	Primary atom site location: structure-invariant direct methods
Least-squares matrix: full	Secondary atom site location: difference Fourier map
*R*[*F*^2^ > 2σ(*F*^2^)] = 0.026	Hydrogen site location: inferred from neighbouring sites
*wR*(*F*^2^) = 0.073	H-atom parameters constrained
*S* = 0.88	*w* = 1/[σ^2^(*F*_o_^2^) + (0.049*P*)^2^] where *P* = (*F*_o_^2^ + 2*F*_c_^2^)/3
1429 reflections	(Δ/σ)_max_ < 0.001
88 parameters	Δρ_max_ = 0.32 e Å^−^^3^
0 restraints	Δρ_min_ = −0.68 e Å^−^^3^

### Special details

**Table:**

Geometry. All e.s.d.'s (except the e.s.d. in the dihedral angle between two l.s. planes) are estimated using the full covariance matrix. The cell e.s.d.'s are taken into account individually in the estimation of e.s.d.'s in distances, angles and torsion angles; correlations between e.s.d.'s in cell parameters are only used when they are defined by crystal symmetry. An approximate (isotropic) treatment of cell e.s.d.'s is used for estimating e.s.d.'s involving l.s. planes.
Refinement. Refinement of *F*^2^ against ALL reflections. The weighted *R*-factor *wR* and goodness of fit *S* are based on *F*^2^, conventional *R*-factors *R* are based on *F*, with *F* set to zero for negative *F*^2^. The threshold expression of *F*^2^ > σ(*F*^2^) is used only for calculating *R*-factors(gt) *etc*. and is not relevant to the choice of reflections for refinement. *R*-factors based on *F*^2^ are statistically about twice as large as those based on *F*, and *R*- factors based on ALL data will be even larger.

### Fractional atomic coordinates and isotropic or equivalent isotropic displacement parameters (Å^2^)

**Table:**

	*x*	*y*	*z*	*U*_iso_*/*U*_eq_	
Cu1	0.5000	0.5000	0.5000	0.04458 (15)	
O1	0.38526 (12)	0.6395 (4)	0.39762 (14)	0.0498 (4)	
O2	0.55742 (13)	0.7912 (3)	0.42774 (14)	0.0498 (4)	
N1	0.40843 (14)	0.8595 (4)	0.34077 (15)	0.0447 (4)	
N2	0.49421 (15)	0.9440 (4)	0.35334 (17)	0.0481 (5)	
C1	0.33006 (17)	1.0104 (6)	0.26151 (19)	0.0506 (5)	
H11	0.3559	1.1716	0.2288	0.061*	
H12	0.2855	1.0778	0.3063	0.061*	
C2	0.2767 (2)	0.8251 (6)	0.1611 (2)	0.0577 (6)	
H21	0.3225	0.7366	0.1232	0.069*	
H22	0.2431	0.6788	0.1928	0.069*	
C3	0.20524 (19)	0.9925 (7)	0.0702 (2)	0.0647 (6)	
H31	0.2394	1.1373	0.0383	0.078*	
H32	0.1606	1.0840	0.1091	0.078*	
C4	0.1491 (3)	0.8157 (8)	−0.0308 (3)	0.0848 (10)	
H41	0.1094	0.6844	0.0000	0.102*	
H42	0.1937	0.7083	−0.0641	0.102*	
C5	0.0852 (3)	0.9888 (11)	−0.1285 (3)	0.1163 (15)	
H51	0.0516	0.8669	−0.1903	0.174*	
H52	0.1242	1.1171	−0.1603	0.174*	
H53	0.0396	1.0918	−0.0966	0.174*	

### Atomic displacement parameters (Å^2^)

**Table:**

	*U*^11^	*U*^22^	*U*^33^	*U*^12^	*U*^13^	*U*^23^
Cu1	0.0460 (2)	0.0446 (2)	0.0432 (2)	−0.0028 (2)	0.01086 (14)	0.0008 (2)
O1	0.0449 (9)	0.0472 (9)	0.0562 (9)	−0.0053 (8)	0.0097 (7)	0.0084 (8)
O2	0.0473 (10)	0.0532 (9)	0.0505 (9)	−0.0062 (8)	0.0147 (7)	0.0020 (7)
N1	0.0485 (12)	0.0428 (11)	0.0428 (9)	−0.0033 (9)	0.0112 (8)	−0.0001 (8)
N2	0.0508 (12)	0.0499 (14)	0.0448 (10)	−0.0036 (9)	0.0137 (8)	0.0016 (8)
C1	0.0521 (13)	0.0470 (11)	0.0533 (11)	0.0052 (14)	0.0138 (10)	0.0037 (14)
C2	0.0538 (15)	0.0578 (16)	0.0584 (14)	0.0037 (13)	0.0073 (12)	−0.0015 (12)
C3	0.0556 (15)	0.0693 (15)	0.0641 (14)	0.0041 (17)	0.0042 (11)	0.0016 (17)
C4	0.069 (2)	0.092 (3)	0.081 (2)	0.0044 (19)	−0.0064 (16)	−0.0107 (19)
C5	0.099 (3)	0.140 (4)	0.086 (2)	−0.002 (3)	−0.025 (2)	−0.002 (3)

### Geometric parameters (Å, º)

**Table:**

Cu1—O1	1.9042 (17)	C2—H21	0.9700
Cu1—O1^i^	1.9042 (17)	C2—H22	0.9700
Cu1—O2^i^	1.9095 (16)	C3—C4	1.512 (4)
Cu1—O2	1.9095 (16)	C3—H31	0.9700
O1—N1	1.325 (3)	C3—H32	0.9700
O2—N2	1.314 (2)	C4—C5	1.521 (5)
N1—N2	1.268 (3)	C4—H41	0.9700
N1—C1	1.461 (3)	C4—H42	0.9700
C1—C2	1.518 (4)	C5—H51	0.9600
C1—H11	0.9700	C5—H52	0.9600
C1—H12	0.9700	C5—H53	0.9600
C2—C3	1.512 (4)		
			
O1—Cu1—O1^i^	180.0	C3—C2—H22	109.4
O1—Cu1—O2^i^	97.54 (7)	C1—C2—H22	109.4
O1^i^—Cu1—O2^i^	82.46 (7)	H21—C2—H22	108.0
O1—Cu1—O2	82.46 (7)	C2—C3—C4	113.1 (3)
O1^i^—Cu1—O2	97.54 (7)	C2—C3—H31	109.0
O2^i^—Cu1—O2	180.0	C4—C3—H31	109.0
N1—O1—Cu1	107.90 (13)	C2—C3—H32	109.0
N2—O2—Cu1	113.09 (14)	C4—C3—H32	109.0
N2—N1—O1	123.09 (19)	H31—C3—H32	107.8
N2—N1—C1	119.6 (2)	C3—C4—C5	112.9 (3)
O1—N1—C1	117.35 (19)	C3—C4—H41	109.0
N1—N2—O2	113.32 (18)	C5—C4—H41	109.0
N1—C1—C2	111.5 (2)	C3—C4—H42	109.0
N1—C1—H11	109.3	C5—C4—H42	109.0
C2—C1—H11	109.3	H41—C4—H42	107.8
N1—C1—H12	109.3	C4—C5—H51	109.5
C2—C1—H12	109.3	C4—C5—H52	109.5
H11—C1—H12	108.0	H51—C5—H52	109.5
C3—C2—C1	111.2 (2)	C4—C5—H53	109.5
C3—C2—H21	109.4	H51—C5—H53	109.5
C1—C2—H21	109.4	H52—C5—H53	109.5

Symmetry code: (i) −*x*+1, −*y*+1, −*z*+1.
